# Multi-Locus Genome-Wide Association Study Reveals the Genetic Architecture of Stalk Lodging Resistance-Related Traits in Maize

**DOI:** 10.3389/fpls.2018.00611

**Published:** 2018-05-07

**Authors:** Yanling Zhang, Peng Liu, Xiaoxiang Zhang, Qi Zheng, Min Chen, Fei Ge, Zhaoling Li, Wenting Sun, Zhongrong Guan, Tianhu Liang, Yan Zheng, Xiaolong Tan, Chaoying Zou, Huanwei Peng, Guangtang Pan, Yaou Shen

**Affiliations:** ^1^Key Laboratory of Biology and Genetic Improvement of Maize in Southwest Region, Maize Research Institute, Sichuan Agricultural University, Chengdu, China; ^2^Research Center of Tumofous Stem Mustard, Chongqing Yudongnan Academy of Agricultural Sciences, Chongqing, China; ^3^Institute of Animal Nutrition, Sichuan Agricultural University, Chengdu, China

**Keywords:** maize, stalk lodging resistance, multi-locus GWAS, QTNs, candidate gene

## Abstract

Stalk lodging resistance, which is mainly measured by stem diameter (SD), stalk bending strength (SBS), and rind penetrometer resistance (RPR) in maize, seriously affects the yield and quality of maize (*Zea mays* L.). To dissect its genetic architecture, in this study multi-locus genome-wide association studies for stalk lodging resistance-related traits were conducted in a population of 257 inbred lines, with tropical, subtropical, and temperate backgrounds, genotyped with 48,193 high-quality single nucleotide polymorphisms. The analyses of phenotypic variations for the above traits in three environments showed high broad-sense heritability (0.679, 0.720, and 0.854, respectively). In total, 423 significant Quantitative Trait Nucleotides (QTNs) were identified by mrMLM, FASTmrEMMA, ISIS EM-BLASSO, and pLARmEB methods to be associated with the above traits. Among these QTNs, 29, 34, and 48 were commonly detected by multiple methods or across multiple environments to be related to SD, SBS, and RPR, respectively. The superior allele analyses in 30 elite lines showed that only eight lines contained more than 50% of the superior alleles, indicating that stalk lodging resistance can be improved by the integration of more superior alleles. Among sixty-three candidate genes of the consistently expressed QTNs, GRMZM5G856734 and GRMZM2G116885, encoding membrane steroid-binding protein 1 and cyclin-dependent kinase inhibitor 1, respectively, possibly inhibit cell elongation and division, which regulates lodging resistance. Our results provide the further understanding of the genetic foundation of maize lodging resistance.

## Introduction

Lodging is one of the most important factors threatening grain yield in maize, and can result in reduced photosynthesis, nutrient transportation, and grain quality (Remison and Dele Akinleye, 1978). The annual yield losses caused by lodging are approximately 5–20% globally (Flintgarcia et al., 2003). In some areas where strong wind and heavy rain occur frequently, the risk of lodging will significantly increase (Adelana, 1980). Some properties of the stem itself are also strongly associated with lodging, such as the structure and mechanical strength of the stem, and the number of vascular bundles (Xu et al., 2017). In addition, Tesso and Ejeta (2011) showed that stalk rot disease can reduce stem strength, which further leads to lodging.

The most direct way to improve breeding populations for quantitative traits is phenotypic selection, where the frequency of favorable alleles is increased within a population over cycles of selection. Previous studies on crop morphological traits showed that plant stem diameter (SD), stalk bending strength (SBS), and rind penetrometer resistance (RPR) are closely associated with stalk lodging in the field (Kashiwagi et al., 2008; Hu et al., 2012, 2013). Furthermore, these three traits are significantly negatively correlated with stalk lodging rate in the field (Ling, 2008). The method for testing RPR involves the use of an electronic rind penetrometer to penetrate the rind of the maize stalk, and the maximum value of penetration is then indicated on the screen of the instrument (Sibale et al., 1992). This method does not affect maize seedling growth.

Genome-Wide Association Study (GWAS) is a very powerful tool for dissecting the genetic basis of complex traits (Korte and Farlow, 2013). To date, GWAS has been used to analyze many agronomic traits such as leaf architecture, maize kernel composition, plant height, oil biosynthesis in maize kernels (Tian et al., 2011; Weng et al., 2011; Cook et al., 2012; Li et al., 2013), and other traits, i.e., Some genetic research on crop lodging has also been conducted using GWAS. Hu et al. (2013) detected a complex polygenic inheritance for SBS-related traits, including the maximum load exerted to breaking (F_max_), the breaking moment (M_max_), and critical stress (σ_max_). A total of seven quantitative trait loci (QTLs) explaining 65.7% of the genotypic variance for these three traits. Ookawa et al. (2010) used chromosome segment substitution lines (CSSL) to identify an effective QTL, *SCM2*, for culm strength in rice, and the near-isogenic line (NIL) carrying *SCM2* showed enhanced culm strength. Moreover, Lin et al. (2005) detected another six QTLs for stem strength, culm wall thickness, pith diameter, and stem diameter using a doubled-haploid (DH) population. Conversely, GWAS for maize lodging has rarely been reported, and the molecular mechanisms of variation for maize lodging-related traits remain poorly understood.

Currently, the Bonferroni correction is applied to control the false positive rate for single-marker GWAS, and some important loci with small effects could be excluded by this stringent correction. Multi-locus GWAS methodologies, such as FASTmrEMMA, ISIS EM-BLASSO, mrMLM, pLARmEB, and FarmCPU, have been shown to effectively resolve this issue. The first four methods have higher power and accuracy for quantitative trait nucleotide (QTN) detection and are more suitable for genetic models (Liu et al., 2016; Wang et al., 2016; Tamba et al., 2017; Wen et al., 2017; Zhang J. et al., 2017). Additionally, a combination of various methods for multi-locus GWAS has also been used to control the false positive rate (Wu et al., 2016; Misra et al., 2017).

Our objectives were to (i) estimate the genetic variance and heritability of SD, SBS, and RPR; (ii) estimate the correlations between these three traits; (iii) detect significant quantitative trait nucleotides (QTNS) for SD, RPR, and SBS in multiple environments; (iv) dissect the genetic basis of variation of lodging-related traits in maize, and (v) identify candidate genes controlling maize stalk lodging-related traits.

## Materials and methods

### Phenotyping of maize lodging-related traits

The SD, SBS, and RPR tests were conducted in an association-mapping panel of 257 diverse inbred lines, which were collected from tropical or subtropical and temperate regions (Li et al., 2013). The names and pedigree information for this association panel are presented in Table S1. The 257 inbred lines were planted in three locations: Xishuangbanna (XSBN, N22°0, E100°79′, Yunnan province, China, 2014), Bijie at Guizhou (GZ, N27°32′, E105°29′, Guizhou province, China, 2014), and Wenjiang (WJ, N30°97, E103°81′, Chengdu, Sichuan province, China, 2014). The 257 inbred lines were sown in a randomized complete block design in two replications. Each plot consisted of a single row (14 plants) that was 3 m in length and 0.75 m from the next row, and the plant density was approximately 62,000 individuals per hectare. Each line was grown in a single-row.

At the flowering stage, 10 plants from each line from each replication were randomly selected for phenotyping and their mean values were computed for the three traits: SD, SBS, and RPR, as detailed in Wang L. M. et al. (2012). Briefly, a Vernier caliper was used to measure the SD (mm) of the 15-cm region above ground. A plant stalk strength appliance SY-S03 with a measuring range from 5 to 500 N and a resolution ratio of 0.1 N (Shijiazhuang Shiya Technology Co., Ltd) was used to measure RPR and SBS, and the units of RPR and SBS are N/mm^2^ and N, respectively.

### Statistical analysis of the phenotype

SPSS version 21.0 (IBM, Armonk, NY, 2012) was used to analyze the phenotypic data, including descriptive statistics (mean, range, standard deviation, skewness, kurtosis) and the correlation analysis. To obtain the best linear unbiased prediction (BLUP) of the three lodging-related traits, the R package lme4 (version 3.4.2, https://www.r-project.org/) was fitted to each genotype: Phenotype ~ (1|Genotype) + (1|Repeat%in%Environment) + (1|Genotype&Environment). Broad-sense heritability (*h*^2^) for each trait was estimated as described by Knapp (Knapp et al., 1985) as: *h*^2^ = σ_*g*_^2^/(σ_*g*_^2^+σ_*gy*_^2^/*r*+σ_*e*_^2^/*yr*), where σ_*g*_^2^, σ_*gy*_^2^, and σ_*e*_^2^ are genetic, genotype-by-environment interaction and residual error variances, respectively, *r* is the number of replications, and *y* is the number of environments. All the variances were calculated using a general linear model in SPSS.

### Genotyping and ML-GWAS

Using publicly available genotypic data from previous studies, all the 257 lines of the association panel were genotyped using the Maize SNP50 BeadChip (Illumine, San Diego, CA), which contains 56,110 SNP loci (Ganal et al., 2011; Yang et al., 2011; Li et al., 2012). A total of 48,193 high-quality SNPs with a minor allele frequency (MAF) ≥0.05 were used in this study (http://www.maizego.org/Resources.html). A total of 500 SNPs for each chromosome were randomly selected to calculate population structure, as described by (Pritchard et al., 2009). Briefly, five independent simulations with 500,000 Markov Chain Monte Carlo (MCMC) replications and 5,000 SNPs were performed with the number of subpopulations (k) ranging from 1 to 12. The results calculated by STRUCTURE software were submitted to the website http://taylor0.biology.ucla.edu/structureHarvester/, and the optimal k was inferred. The relative kinship (K matrix) between the lines was calculated as previously described in Wang et al. (2016) and Zhang J. et al. (2017). Four multi-locus GWAS methods including mrMLM, FASTmrEMMA, pLARmEB, and ISIS EM-BLASSO were used in this study. All parameters were set at default values (Wang et al., 2016; Tamba et al., 2017; Wen et al., 2017; Zhang J. et al., 2017).

### Annotation of candidate genes and pathway enrichment analysis

Those genes with common SNPs in the GWAS result were selected as candidate genes. The maize inbred line B73 assembly v2 that was used as the reference genome for the candidate gene analyses was publicly available on the MaizeGDB genome browser (Andorf et al., 2010). The methods of Kyoto Encyclopedia of Genes and Genomes (KEGG) pathways for these candidate genes were annotated as described by Zhang Y. et al. (2017).

## Results

### Diversity and heritability of the three lodging-related traits

**Figure 1:**
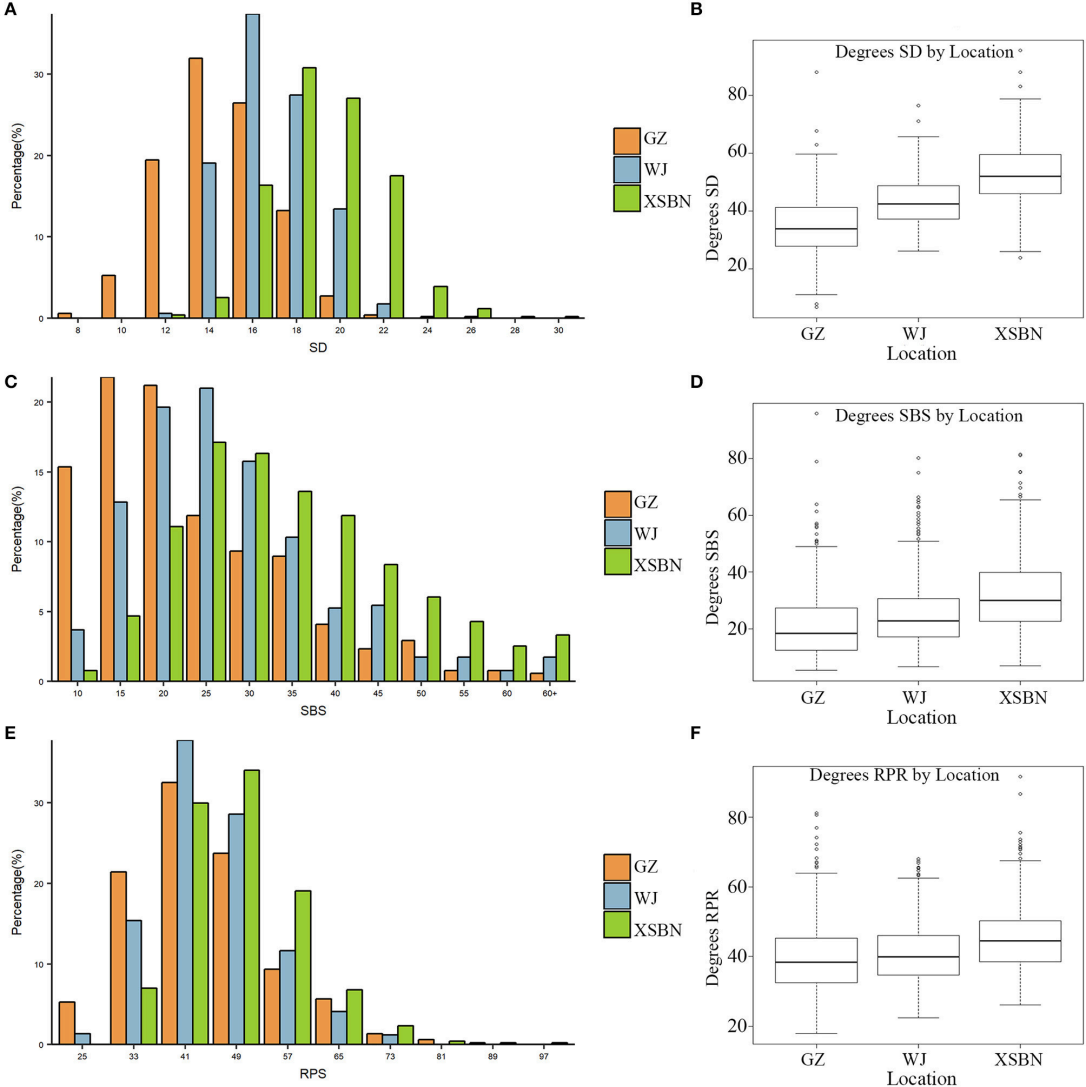
Frequency distributions of SD **(A)**, SBS **(C)**, RPR **(E)** in 257 maize inbred lines and the boxplots for SD **(B)**, SBS **(D)**, RPR **(F)** in the three environments.

The phenotypic characteristics for SD, SBS, and RPR across the three environments are shown in Table 1 and Figure 1. As shown in Table 1, the skewness and kurtosis were less than 1 for SD and RPR, indicating that SD and RPR followed a normal distribution. SBS was slightly skewed to the left (Figures 1A,C,E). For the above three traits, the means of phenotypic values decreased from XSBN, WJ, to GZ; the coefficients of variation ranged from 11.87~14.2, 36.42~49.29, and 19.43~23.45 (%), respectively (Table 1, Figures 1B,D,F).

**Table 1:** Phenotypic performance of the three lodging resistance-related traits in 257 inbred lines under three environments.

**Trait**	**Env**.	**Mean**	**Range**	**SDD**	**CV (%)**	**Skew**	**Kurt**
SD	GZ	13.62	9.23–19.44	1.93	14.20	0.43	−0.16
	WJ	15.80	12.03–21.56	1.88	11.87	0.40	−0.26
	XSBN	18.19	12.11–25.19	2.22	12.18	0.20	−0.06
SBS	GZ	21.26	6.43–64.94	10.48	49.29	1.22	1.65
	WJ	25.33	7.11–70.58	10.68	42.17	1.15	1.77
	XSBN	32.25	7.91–70.84	11.74	36.42	0.70	0.32
RPR	GZ	39.86	20.24–73.79	9.35	23.45	0.73	0.62
	WJ	41.07	23.90–67.46	7.98	19.43	0.64	0.22
	XSBN	45.03	27.54–79.79	8.87	19.70	0.82	1.39

*SD (stalk diameter) is measured in the unit of millimeter (mm), SBS (stalk bending strength) is measured in the unit of newton (N) and RPR (rind penetrometer resistance) is measured in the unit of newton per square millimeter (N/mm^2^)*.

*Env. Represents environments; GZ, WJ, and XSBN represent Guizhou, Wenjiang and Xishuangbanna, respectively. SDD, standard deviation*.

*CV, coefficient of variation*.

The results of correlation analysis were showed in Table 2. Significant correlations between the traits across three environments were observed. For example, the correlation coefficients between SD and SBS in GZ, WJ, and XSBN were 0.762, 0.615 and 0.668 (*P*-values <0.01), respectively; the correlations between SD and RPR across three environments were relatively smaller (0.219 < *r* < 0.308, *P* < 0.01) than those between SBS and RPR (0.507 < *r* < 0.652, *P* < 0.01). In addition, a significant correlation between different environments was observed for each of the three traits (Table 2).

**Table 2:** Phenotypic correlation coefficients between lodging resistance-related traits across three environments.

**Trait**		**SD**			**SBS**			**RPR**		
	**Environment**	**GZ**	**WJ**	**XSBN**	**GZ**	**WJ**	**XSBN**	**GZ**	**WJ**	**XSBN**
SD	GZ	1								
	WJ	0.314^**^	1							
	XSBN	0.356^**^	0.568^**^	1						
SBS	GZ	0.762^**^	0.297^**^	0.255^**^	1					
	WJ	0.349^**^	0.615^**^	0.319^**^	0.524^**^	1				
	XSBN	0.300^**^	0.387^**^	0.668^**^	0.385^**^	0.485^**^	1			
RPR	GZ	0.219^**^	0.238^**^	0.131^*^	0.507^**^	0.381^**^	0.352^**^	1		
	WJ	0.078	0.308^**^	0.099	0.324^**^	0.652^**^	0.307^**^	0.661^**^	1	
	XSBN	0.036	0.279^**^	0.274^**^	0.283^**^	0.391^**^	0.614^**^	0.688^**^	0.644^**^	1

*,** *Indicate significance level at P < 0.05 and 0.01, respectively*.

*Env. Represents environments; GZ, WJ, and XSBN represent Guizhou, Wenjiang, and Xishuangbanna, respectively*.

In the analysis of variance for the three traits, highly significant variations for genotypes (G) and environments (E) and significant variation for genotype-by-environment interaction were found (Table 3). This indicates the important roles of both environment and G × E interaction. The broad-sense heritabilities (*h*^2^) for SD, SBS, and RPR across the three environments in the 257 inbred lines ranged from 0.679 (SD) to 0.854 (RPR), indicating the predominant role of genetic factors for these traits (Table 3).

**Table 3:** Analysis of variance (ANOVA) for lodging resistance-related traits of 257 lines in three environments.

**Trait**	**Source of variation**	**Mean square**	***F***	**Significance**	***H*^2^**
SD	Environment (E)	2,679.898	1,046.302	<0.01^**^	0.679
	Genotype (G)	14.811	5.783	<0.01^**^	
	Replication	9.878	3.857	0.051	
	G × E	4.761	1.859	<0.01^**^	
	Residual Error	2.561		<0.01^**^	
SBS	Environment (E)	15,870.661	288.548	<0.01^**^	0.720
	Genotype (G)	463.779	8.432	<0.01^**^	
	Replication	35.159	0.639	0.424	
	G × E	129.899	2.362	<0.01^**^	
	Residual Error	55.002		<0.01^**^	
RPR	Environment (E)	3,761.979	127.496	<0.01^**^	0.854
	Genotype (G)	355.790	12.058	<0.01^**^	
	Replication	123.123	4.173	0.042^*^	
	G × E	51.886	1.758	<0.01^**^	
	Residual Error	29.507		<0.01^**^	

*,** *Indicate significance level at P < 0.05 and 0.01, respectively*.

### QTNs identified by ML-GWAS

The Δ*K* calculation of STRUCTURE indicated a peak (*K* = 2) in the broken line graph reflecting the number of subpopulations (K) (Figures S1A,B), indicating that the 257 maize inbred lines could be divided into two subpopulations. Owning to significant variations for each of the three lodging-related traits in 257 maize inbred lines across the three locations, BLUP values across the three locations were also used for the GWAS. In total, 423 significant QTNs were identified at the critical logarithm of odds (LOD) score (≥3) for these traits in the three environments using mrMLM, FASTmrEMMA, PLARmEB and ISIS EM-BLASSO (Table S2, Figure S2).

A total of 126 significant QTNs, mainly distributed on chromosomes 1, 2, 3, 5, 6, 8, and 9, were detected to be associated with SD (Table S2, Figure S2A). Among them, 29 QTNs were common across the methods or the locations. The LOD of these 32 QTNs identified by mrMLM ranged from 3.03 to 6.25, and the percentage of phenotypic variation explained by each QTN (PVE) in GZ, WJ, XSBN, and BLUP was 30.96, 40.90, 44.21, and 54.38 (%), respectively. The LOD scores of the significant 21 QTNs identified by FASTmrEMMA ranged from 3.08 to 6.21, and the PVE in GZ, WJ, XSBN, and BLUP for SD was 19.51, 20.51, 22.31, and 21.25 (%), respectively. For PLARmEB, the LOD scores of the 43 QTNs ranged from 3.01 to 7.83 in GZ, WJ, XSBN, and BLUP, and PVE was 13.84, 35.20, 31.17, and 36.84 (%), respectively. The LOD scores of the 66 QTNs detected by ISIS EM-BLASSO ranged from 3.00 to 14.08, and the PVE in GZ, WJ, XSBN and BLUP was 51.15, 41.37, 48.99, and 44.37 (%), respectively.

In total, 148 significant QTNs were correlated with SBS, and were evenly distributed on 10 chromosomes under the environments and BLUP model. Among them, 35 QTNs were common across the methods or the locations. The LOD values of the 148 QTNs identified by mrMLM, FASTmrEMMA, PLARmEB, and ISIS EM-BLASSO were in the range of 3.01~8.78, 3.32~10.75, 3.09~8.69, and 3.05~12.18, respectively (Table S2, Figure S2B). Among these QTNs, 43 identified by mrMLM explained 58.31, 53.47, 52.02, and 57.69 (%) of the phenotypic variation in GZ, WJ, XSBN, and BLUP for SBS, respectively. Conversely, 26.98, 43.87, 17.02, and 26.10 (%) of the phenotypic variation was separately explained by 28 QTNs using FASTmrEMMA. Using PLARmEB, the PVE was 28.77, 30.40, 17.31, and 49.24 (%) in the different environments, respectively. The PVE in GZ, WJ, XSBN, and BLUP for SBS was 53.88, 64.19, 56.12, and 49.64 (%), respectively, for the 73 QTNs by ISIS EM-BLASSO.

We detected a total of 149 RPR-associated QTNs with LODs ranging from 3.01 to 14.39 in the three environments and BLUP model, and were mainly located on chromosomes 1, 2, 4, 5, 7, 8, and 9 (Table S2, Figure S2C). And 47 QTNs were common across the methods or the locations. Among these, four QTNs were also detected in SBS traits. Of the 149 RPR-associated QTNs, 54, 31, 57, and 74 QTNs were separately identified by mrMLM, FASTmrEMMA, PLARmEB, and ISIS EM-BLASSO, which explained 60.91~67.76, 23.53~35.38, 30.90~56.86, and 45.28~63.77 (%) of the phenotypic variation, respectively.

### Verification of the common QTNs by multi-methods or across environments

**Figure 2:**
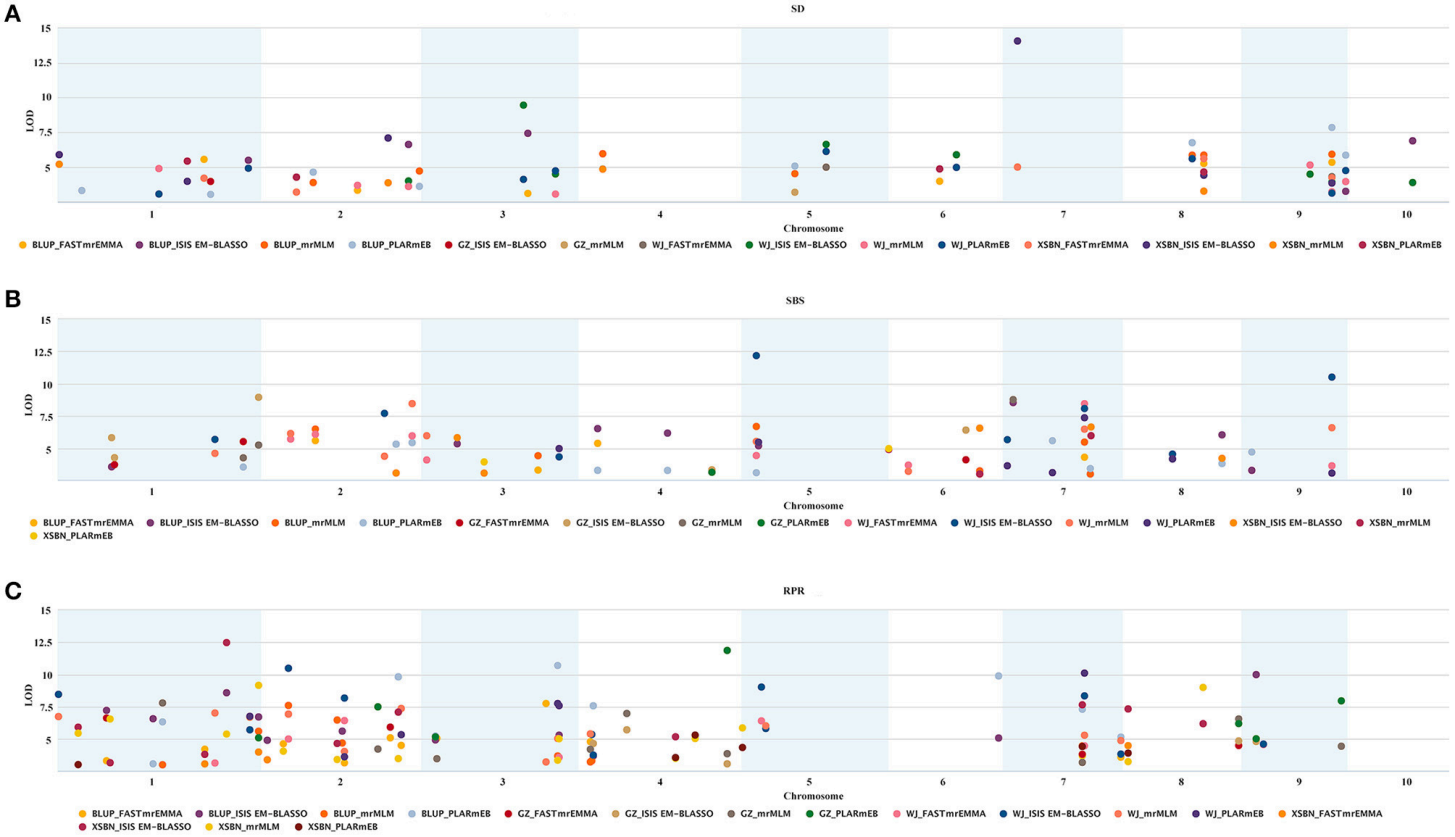
Repeatability and significance of the SNPs associated with the three lodging resistance-related traits in the three environments and BLUP. The significance threshold is LOD = 3.0. **(A–C)** Represent SD, SBS, and RPR, respectively.

A total of 107 QTNs were co-identified by at least two of the methods or across different environments, among which 29, 34, and 48 were associated with SD, SBS, and RPR, respectively (Table S3 and Figure 2). To verify the significance of each common QTN, we divided the population into two groups according to their allele types and compared the mean phenotypic values between the two groups. For SD, the average of the group containing the superior alleles was significantly greater than the group containing inferior alleles, with the exception of the SNPs SYN35339, SYN6428, PZE-102085765, and PZE-101121408 (Table S4). As for SBS and RPR, the group with the superior alleles showed a significantly larger mean than the group with inferior alleles for every common SNP (Table S4). These results verified the reliability and significance of the common QTNs identified by these ML-GWAS methods.

### Utilization of superior alleles in elite maize lines

**Figure 3:**
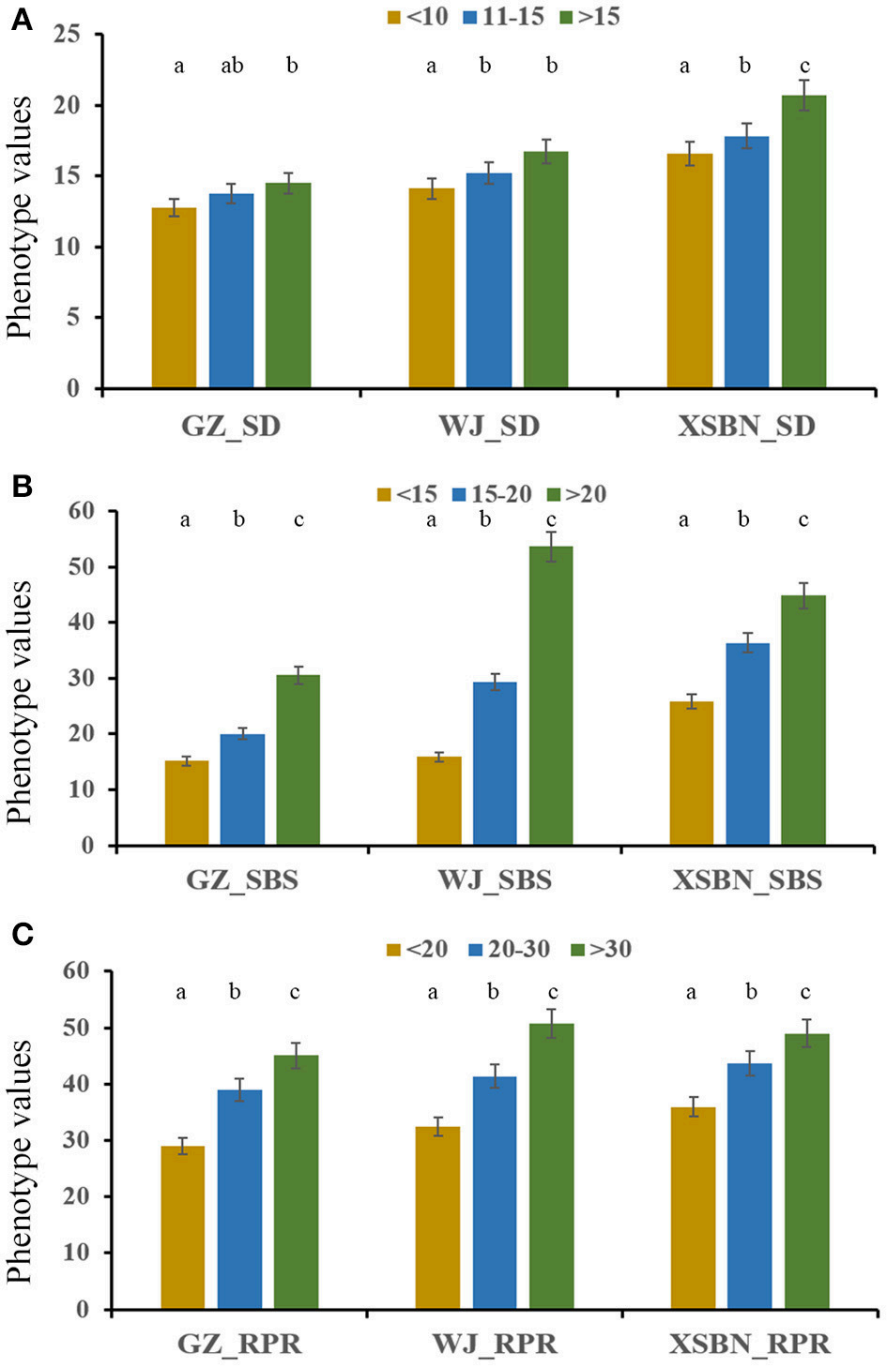
The phenotypic values in the maize elite inbred lines with different numbers of superior alleles for SD **(A)**, SBS **(B)**, and RPR **(C)**.

**Figure 4:**
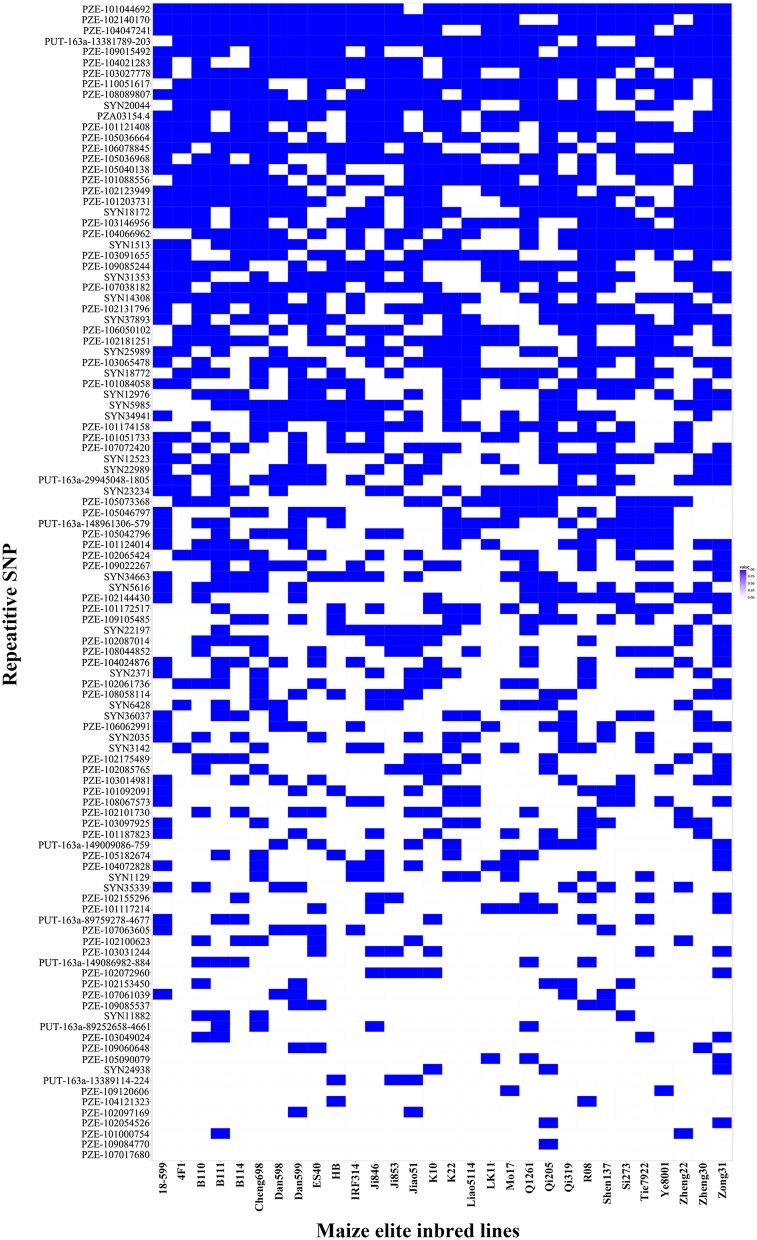
The superior allele SNP distributions in the 30 maize elite inbred lines. Blue and white colors represent superior and inferior alleles, respectively.

Thirty elite inbred lines from China and America that have excellent agronomic traits and serve as the parents of commercialized hybrid varieties were included in the maize population, which enabled us to evaluate the utilization of the superior alleles for maize breeding. The results indicated that the percentage of SD superior alleles in the elite lines ranged from 27.59 to 55.17% (Table S5). The lines with >15 superior alleles indicated a significantly higher SD phenotypic value, with an average of 14.50 in GZ, 16.75 in WJ, and 20.72 in XSBN, whereas the lines with 0~10 superior alleles had average SD values of 12.78, 14.13, and 16.61 in GZ, WJ, and XSBN, respectively (Table S5, Figure 3A). The utilization of the SBS superior alleles in the elite lines ranged from 25.71 to 65.71% (Table S5). The phenotypic averages of the lines with >20 superior alleles were 30.56, 53.68, and 44.83 in GZ, WJ, and XSBN, respectively, whereas those with 15~20 superior alleles had a lower average of 15.14, 15.87, and 25.85 in GZ, WJ, and XSBN, respectively (Table S5, Figure 3B). As for RPR, the elite lines contained 29.17~66.67% of the superior alleles (Table S5). The average RPR in the lines with >30 superior alleles were 45.06, 50.69, and 49.01 in GZ, WJ, and XSBN, respectively; however, those lines with <20 superior alleles had average RPR values of 29.05, 32.43, and 35.97 in GZ, WJ, and XSBN, respectively (Table S5, Figure 3C). The results suggested that these superior alleles had an additive effect on the lodging resistance-related traits. Further analysis indicated that only eight of the 30 elite inbred lines had more than 50% utilization of all the superior alleles (Table S5, Figure 4), implying that the superior alleles were not efficiently selected during maize breeding. In future work, integrated utilization of the superior alleles would be an efficient approach for lodging-resistance breeding in maize by marker-assisted selection (MAS).

### Candidate genes associated with common QTNs

To further understand the molecular basis of lodging-related traits, we focused on the candidate genes that were directly associated with the common QTNs. As a result, 19, 17, and 30 candidate genes around their common QTNs were found to be associated with SD, SBS, and RPR, respectively. The annotations for the candidate genes are displayed in Table S3, with seven transcription factors, eight kinase-related proteins, and four transport proteins involved. These genes mainly participate in metabolic pathway, genetic information processing, environmental information processing, cellular processes, and organismal systems (Table 4).

**Table 4:** SNPs, chromosomal position and the pathway of candidate genes significantly associated with three lodging resistance-related traits identified by multi GWAS methods across all environments.

**SNPs**	**Traits**	**Genes**	**Genotype**	**Chr**	**Gene interval (bp)**	**Annotation**	**Pathway class A**	**Pathway class B**
PZE-103065478	SBS	GRMZM2G018447	A/G	3	93187188~93205169	Ubiquitin-conjugating enzyme 15	Genetic Information Processing	Folding, sorting and degradation
PZE-105090079	SD	GRMZM2G038126	A/G	5	125802058~125817596	26S protease regulatory subunit 6B homolog	Genetic Information Processing	Folding, sorting and degradation
PZE-104021283	RPR	GRMZM2G047800	A/G	4	22835928~22842145	NAD(P)-binding Rossmann-fold superfamily protein	Metabolism	Metabolism of terpenoids and polyketides
SYN37893	RPR	GRMZM2G051101	T/G	4	219995778~219999760	Putative seven in absentia domain family protein	Genetic Information Processing	Folding, sorting and degradation
SYN20044	RPR	GRMZM2G058584	A/G	1	285124791~285129572	Histidinol dehydrogenase chloroplastic	Metabolism	Amino acid metabolism
							Metabolism	Global and Overview
PZE-102123949	RPR	GRMZM2G067514	A/G	2	172399126~172403574	Phosphoglycerate mutase family protein	Genetic Information Processing	Folding, sorting and degradation
							Genetic Information Processing	Transcription
PZE-102144430	RPR	GRMZM2G083504	A/G	2	191378248~191380615	Transcription factor bHLH62	Environmental Information Processing	Signal transduction
							Organismal Systems	Environmental adaptation
SYN1129	RPR	GRMZM2G084181	A/G	2	9114721~9126810	ABC transporter C family member 2	Environmental Information Processing	Membrane transport
PZE-104047241	RPR	GRMZM2G103721	A/G	4	71631328~71640766	Phosphatidylinositol 3-kinase VPS34	Cellular Processes	Transport and catabolism
							Environmental Information Processing	Signal transduction
							Metabolism	Carbohydrate metabolism
PZE-101172517	SD	GRMZM2G119357	T/C	1	216471619~216479224	Chromatin remodeling protein EBS	Cellular Processes	Transport and catabolism
							Genetic Information Processing	Folding, sorting and degradation
							Genetic Information Processing	Transcription
SYN34663	RPR	GRMZM2G135341	A/G	7	174359079~174365275	BADH-like protein	Metabolism	Amino acid metabolism
SYN18172	RPR	GRMZM2G138255	T/G	8	8857140~8860877	ARM repeat superfamily protein	Cellular Processes	Transport and catabolism
SYN11882	SBS	GRMZM2G155312	A/G	1	275140650~275150229	Leucine-rich repeat protein kinase family protein	Organismal Systems	Environmental adaptation
PZE-107017680	SBS	GRMZM2G156692	T/G	7	15316520~15323155	proline-rich family protein	Genetic Information Processing	Transcription
SYN2035	SBS	GRMZM2G304638	A/G	1	297169418~297180338	BEACH domain-containing protein C2	Metabolism	Amino acid metabolism
							Metabolism	Global and Overview
PZE-101203731	RPR	GRMZM2G324276	T/C	1	250294172~250296186	Core-2/I-branching beta-16-N-acetylglucosaminyltransferase family protein	Cellular Processes	Transport and catabolism
PZE-108089807	SBS	GRMZM2G375975	T/C	8	146830973~146836666	Putative MAP kinase family protein	Human Diseases	Endocrine and metabolic diseases
SYN5616	RPR	GRMZM2G431309	A/G	2	207837259~207841334	GRAS transcription factor	Environmental Information Processing	Signal transduction

## Discussion

According to previous studies, the strength of the maize stalk depends on the tissue and morphology, and the morphology of the stalk is largely determined by the mechanical stresses in maize (Von et al., 2015). SD, SBS, and RPR were demonstrated to show potential as selective breeding indexes for improving lodging resistance (Liu et al., 2011; Xiang et al., 2016). The heritability and genetic models vary among different studies since the calculations depend on the experimental populations, design, and conditions (Lynch and Walsh, 1998). The genetic architecture of lodging resistance-related traits has been illustrated in diverse maize populations by linkage mapping. (Kashiwagi et al., 2008; Hu et al., 2012, 2013). However, the genetic basis and the molecular pathways underlying lodging resistance-related traits, as well as the major genes associated with the traits, remain largely unknown. In this study, we interpreted the natural variation and revealed the genetic architecture of three lodging resistance-related traits based on 257 maize inbred lines by ML-GWAS analysis. And identified the candidate genes and their possible pathways for stalk lodging resistance.

### Genetic basis of lodging-related traits

In this study, the three lodging-related traits exhibited wide phenotypic variation and were normally distributed. ANOVA showed that the genetic effects and interactive effects between the genetics and environment were both significant for these traits, and the heritability (*h*^2^) was very high for SD, SBS, and RPR. Previous studies on SD in different crops mainly focused on the phenotypic correlations with stalk mechanical strength and the identification of QTLs for SD, whereas the heritability of SD has not been investigated (Lin et al., 2005; Kashiwagi et al., 2008). In our study, *h*^2^ was 0.679 across the three environments for SD. The *F*_max_, *M*_max_, and σ_max_ can be used as tools to determine SBS accreditation (Timoshenko and Gere, 1972). An *h*^2^ of 0.84 for *F*_max_, was reported in rice, and in maize the *h*^2^ for *F*_max_, *M*_max_, and σ_max_ were 0.81, 0.79, and 0.75, respectively (Sun, 1987; Hu et al., 2013). Both these estimates are in close agreement with the estimates of SBS (*h*^2^ = 0.720) in our study (Table 3). The *h*^2^ estimates were previously found to range from 0.81 to 0.92 for RPR in different maize populations (Flintgarcia et al., 2003; Hu et al., 2012), which corroborates our value of 0.854 across the three environments. In combination with previous results, our findings suggest that all the measured lodging-related traits showed high precision and that the three lodging resistance-related traits generally exhibited high heritability.

Phenotypic correlations were observed among the three lodging-related traits. For instance, the correlation coefficient between SBS and RPR was 0.507 in GZ, 0.652 in WJ, and 0.614 in XSBN, respectively (Table S2). Meanwhile, we identified four QTNs (PZE-101187823, SYN31353, PZE-105036664, and PZE-107063605), all of which were associated with both SBS and RPR (Table S3). The above results suggested that some genetic factors were shared among these lodging resistance-related traits.

### Common candidate genes reveal the possible molecular basis of lodging resistance

No previous studies have reported on GWAS for SD, SBS, and RPR in maize. However, some studies have evaluated the QTLs. Hu et al. (2013) detected two, three, and two QTLs for F_max_, M_max_, and σ_max_, respectively, using 216 recombinant inbred lines and 129 SSR markers. Among them, a QTL of σ_max_, an important parameter for characterizing SBS, was located in markers umc1993 and bulg1450. In the present research, a QTN on Chr10 (position: 137282081 bp) for SBS locates exactly in the interval of the σ_max_ QTL reported by Hu et al. (2013) (Table S2). The remaining QTNs in the present study are the first to be reported as associated with lodging resistance-related traits in maize.

Furthermore, we identified 63 common candidate genes in total that were around common QTNs for lodging-related traits. Notably, GRMZM5G856734 encodes Membrane steroid-binding protein 1 (MSBP1) (Table S3), whose homologous gene *MSBP1* in *Arabidopsis thaliana* was proven to be involved in the inhibition of cell elongation (Yang et al., 2005). Interestingly, the candidate gene GRMZM2G116885 that encodes cyclin-dependent kinase inhibitor 1 was associated with both SBS and RPR. The homologous gene of GRMZM2G116885 in *Arabidopsis* was reported to be involved in coordinated cell growth or cell division (Bemis and Torii, 2007). It is generally known that cell elongation and cell wall thickening regulate plant lodging resistance (Fan et al., 2017). According to RNA-Seq data from the previous study, the candidate genes GRMZM5G856734 and GRMZM2G116885 had high expression levels in maize stems, with the FPKM are 115.5 and 58.0, respectively (Sekhon et al., 2012). In addition, more than 90% of the candidate genes found in our study were expressed in maize stems, especially the expression levels of GRMZM2G038126, GRMZM2G073934, GRMZM2G058584, and GRMZM2G084181 were extremely high (Table S3). The functional validation of these genes should be addressed in future work.

Additionally, seven candidate genes were classified into transcription factors based on their functional annotation, including ethylene-responsive transcription factor 12, bHLH-transcription factor 105, bHLH-transcription factor 65, GRAS transcription factor, transcription factor VOZ1, and MYB 9 transcription factor (Table S3). Transcription factors are a group of proteins that regulate targeted gene expression in particular cells at a certain time, and are vital for cell division, growth, and death (Latchman, 1997; Riechmann and Meyerowitz, 1998; Guilfoyle and Hagen, 2007).

### The superiority of the new ML-GWAS

Previous studies demonstrated that the single-locus GWAS was useful to dissect complex agronomic trait by using general linear models (GLMs) and mixed linear models (MLMs) (Zhang et al., 2010; Wang M. et al., 2012). High false positive rates are an obvious shortcoming for GLMs, because there is no kinship among materials as covariate (Pace et al., 2015). The screening criteria of significance for single-locus GWAS is *P* = 0.05/*m* (*m* is the number of markers) (Perneger, 1998). Owning to large number of SNPs (Gordon et al., 2016), some important loci might be excluded under the stringent criteria in MLM. To remedy the shortcomings of the methods mentioned above, ML-GWAS methods have recently been explored, including mrMLM (Wang et al., 2016), pLARmEB (Zhang J. et al., 2017), ISIS EM-BLASSO (Tamba et al., 2017), and FASTmrEMMA (Wen et al., 2017). Several studies have individually analyzed published data using the multi-locus methods, and have indicated that these methods constituted effective approaches with high detection power and less stringent criteria (Wang et al., 2016; Tamba et al., 2017; Wen et al., 2017; Zhang J. et al., 2017). In our study, a total of 126, 77, 176, and 230 significant QTNs were detected for three lodging-related traits using mrMLM, FASTmrEMMA, pLARmEB, and ISIS EM-BLASSO, respectively (Figure S2, Table S2). A comparison of the four methods showed that ISIS EM-BLASSO was more powerful than the other three multi-locus methods in the identification of QTNs for lodging resistance-related traits (Table S2, Figure S2). Furthermore, some stably expressed QTNs were detected in the multi-environment and BLUP model using multi-methods (Tables s2, s3). Notably, the candidate genes GRMZM5G856734 and GRMZM2G116885, were proven to inhibit cell elongation and division, which regulates lodging resistance. However, only 4, 4, and 7 SNPs were detected for SD, SBS, and RPR, respectively, from FarmCPU (R packages FarmCPU, K and PCA calculated by SPAGeDi software and GAPIT package, respectively. The threshold is *P*-value = 0.05/48193) (Table S6). In addition, six of these SNPs were also be detected by ML-GWAS methods. Using GAPIT (R packages GAPIT) method, only one SD-associated SNP was found in XSBN, which was also detected in the ML-GWAS methods (Table S6). Our study demonstrated that improved efficiency and accuracy could be achieved by a combination of the new multi-locus methods for identification of lodging resistance-related QTNs in maize.

## Conclusions

SD, SBS, and RPR were used in this study to dissect the genetic basis of stalk lodging resistance in maize using ML-GWAS methods. Among all the significantly associated QTNs for the three traits, 107 were commonly identified across multiple methods or environments. Around these common QTNs, sixty-three candidate genes were responsive for maize lodging resistance. These QTNs provide the important information for the marker-assisted selection, and these candidate genes should improve our understanding of the molecular basis of maize lodging resistance.

## Author contributions

YS and PL designed the experiments. YLZ, XZ, QZ, MC, FG, ZL, WS, and YZ performed the analysis. YLZ, YS, ZG, TL, YZ, XT, CZ, HP, and GP drafted the manuscript. All the authors critically revised and approval the final version of this manuscript.

### Conflict of interest statement

The authors declare that the research was conducted in the absence of any commercial or financial relationships that could be construed as a potential conflict of interest.

## Supplementary material

Figure S1 Population structure of the 257 maize inbred lines based on 48,193 SNP markers. **(A)** Plot of delta.K against putative K ranging from 1 to 12. **(B)** Stacked bar plot of ancestry relationship of the natural population.

Figure S2 Manhattan plots showing all the significant SNPs associated with lodging resistance-related traits using four ML-GWAS methods across three environments and BLUP. **(A–C)** represent SD, SBS, and RPR, respectively. Points of different colors represent different methods and environments.

Table S1 Pedigree information of the maize accessions used in this study.

Table S2 ML-GWAS detected significant signals associated with SD, SBS, and RPR across the three environments and BLUP.

Table S3 Repetitive SNPs and their information by ML-GWAS consistently identified in multiple methods or environments.

Table S4 Distribution of the important SNPs superior alleles in the 257 inbred lines.

Table S5 Distribution and utilization percentage of the important SNPs superior alleles in the 30 maize elite inbred lines.

Table S6 Significant signals associated with SD, SBS, and RPR, detected by FarumCPU and GAPIT.
