# Supplementary figures and images for: Multi-Locus Genome-Wide Association Study Reveals the Genetic Architecture of Stalk Lodging Resistance-Related Traits in Maize
Source: Front Plant Sci. 2018 May 7;9:611. doi: 10.3389/fpls.2018.00611 (PMC5949362; doi:10.3389/fpls.2018.00611)

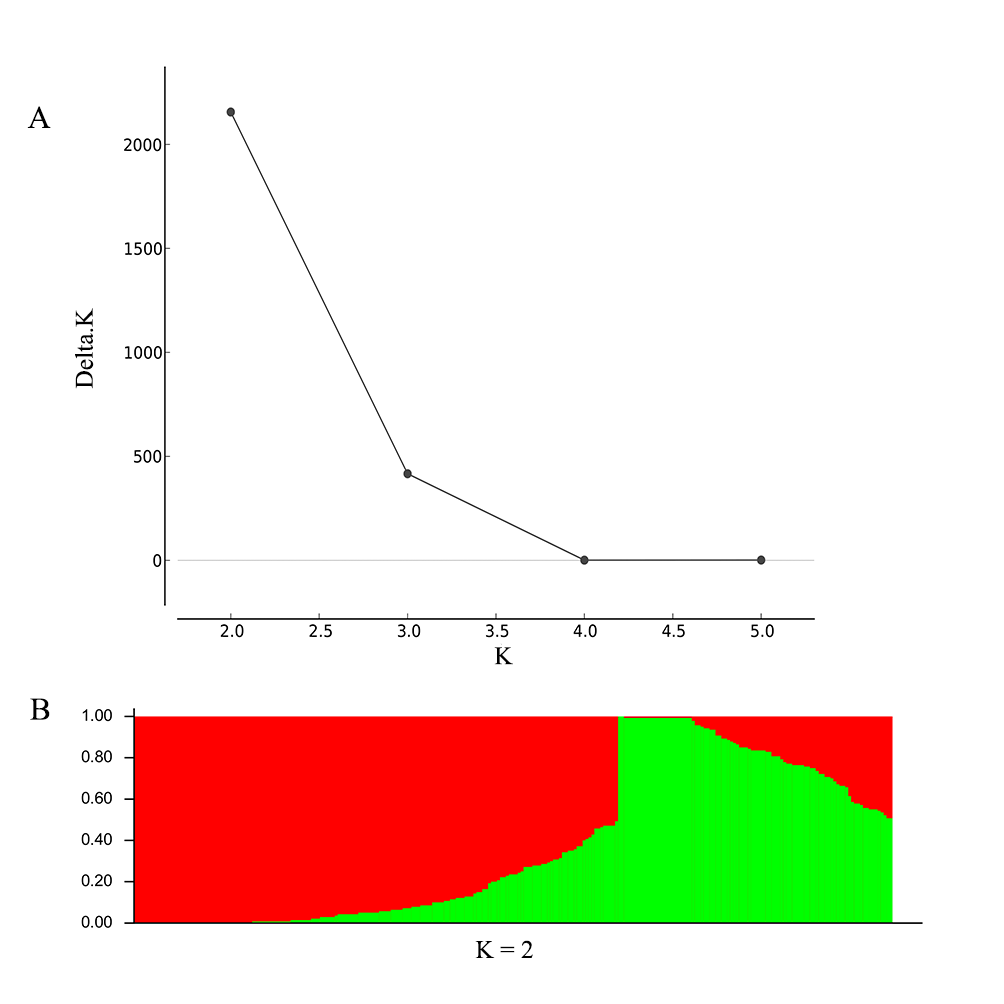

Supplement: Figure S1 — Population structure of the 257 maize inbred lines based on 48,193 SNP markers. (A) Plot of delta.K against putative K ranging from 1 to 12. (B) Stacked bar plot of ancestry relationship of the natural population. [file Image_1.TIF]

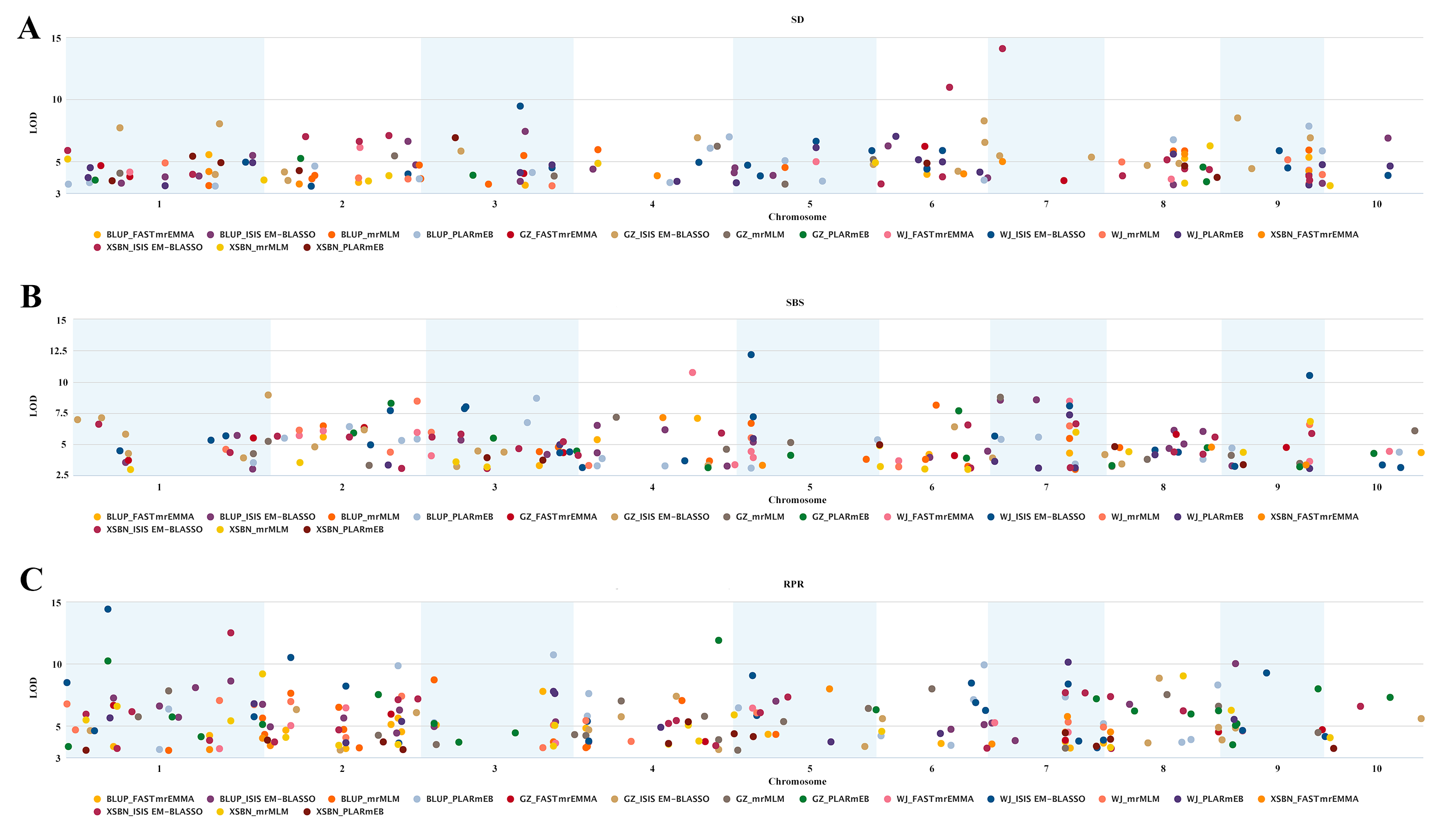

Supplement: Figure S2 — Manhattan plots showing all the significant SNPs associated with lodging resistance-related traits using four ML-GWAS methods across three environments and BLUP. (A–C) represent SD, SBS, and RPR, respectively. Points of different colors represent different methods and environments. [file Image_2.TIF]
