# Supplementary material for: Multi-Locus Genome-Wide Association Study Reveals the Genetic Architecture of Stalk Lodging Resistance-Related Traits in Maize
Source: Front Plant Sci. 2018 May 7;9:611. doi: 10.3389/fpls.2018.00611 (PMC5949362; doi:10.3389/fpls.2018.00611)
Supplement: Table S4 — Distribution of the important SNPs superior alleles in the 257 inbred lines. [file Table_4.DOCX]

**Table S4** The distribution of the important SNPs superior alleles in the 257 inbred lines.

|  |  |  | Allele type | | Phenotype value (mean) | | | | | | Allele number | | Allele percentage (%) | |
| --- | --- | --- | --- | --- | --- | --- | --- | --- | --- | --- | --- | --- | --- | --- |
| Trait | SNPname | CHR | Superior | Inferior | GZ_Superior | GZ_Inferior | WJ_Superior | WJ_Inferior | XSBN_Superior | XSBN_Inferior | Superior | Inferior | Superior | Inferior |
| SD | PUT-163a-13389114-224 | 1 | A | G | 14.33 | 13.49 | 16.15 | 15.74 | 18.59 | 18.15 | 35 | 217 | 13.62% | 84.44% |
|  | PZE-101051733 | 1 | G | A | 13.83 | 13.43 | 16.09 | 15.34 | 18.49 | 17.7 | 145 | 100 | 56.42% | 38.91% |
|  | SYN22197 | 2 | A | C | 14.12 | 13.2 | 16.3 | 15.43 | 18.48 | 17.94 | 106 | 143 | 41.25% | 55.64% |
|  | PZE-102175489 | 2 | C | A | 14.24 | 13.46 | 16.52 | 15.62 | 19.16 | 17.92 | 53 | 198 | 20.62% | 77.04% |
|  | PUT-163a-89759278-4677 | 3 | A | T | 13.55 | 13.5 | 16.4 | 15.63 | 18.22 | 18.22 | 44 | 177 | 17.12% | 68.87% |
|  | PZE-103091655 | 3 | G | A | 13.75 | 13.3 | 16.08 | 15.15 | 18.23 | 17.89 | 175 | 66 | 68.09% | 25.68% |
|  | PZE-103097925 | 3 | A | C | 14.4 | 13.33 | 16.27 | 15.64 | 18.76 | 17.95 | 65 | 182 | 25.29% | 70.82% |
|  | SYN14308 | 4 | A | G | 13.76 | 13.3 | 16.08 | 15.31 | 18.57 | 17.04 | 185 | 45 | 71.98% | 17.51% |
|  | PZE-105073368 | 5 | C | A | 13.92 | 13.12 | 15.97 | 15.49 | 18.28 | 18 | 166 | 87 | 64.59% | 33.85% |
|  | SYN34941 | 6 | A | G | 13.94 | 12.98 | 16.09 | 15.36 | 18.53 | 17.58 | 150 | 66 | 58.37% | 25.68% |
|  | PUT-163a-149086982-884 | 7 | A | C | 13.62 | 13.46 | 15.81 | 15.79 | 17.86 | 18.27 | 39 | 194 | 15.18% | 75.49% |
|  | SYN12976 | 8 | G | A | 13.94 | 13.34 | 16 | 15.62 | 18.65 | 17.77 | 116 | 134 | 45.14% | 52.14% |
|  | PZE-108058114 | 8 | G | A | 14.27 | 13.23 | 16.06 | 15.65 | 18.66 | 17.9 | 89 | 158 | 34.63% | 61.48% |
|  | PZE-109060648 | 9 | G | A | 13.71 | 13.55 | 16.64 | 15.48 | 18.55 | 18.05 | 61 | 188 | 23.74% | 73.15% |
|  | PZE-109084770 | 9 | A | G | 14.14 | 13.5 | 16.85 | 15.61 | 19.42 | 17.94 | 36 | 203 | 14.01% | 78.99% |
|  | PZE-109085244 | 9 | A | G | 13.87 | 13.16 | 16.11 | 15.2 | 18.36 | 17.8 | 160 | 84 | 62.26% | 32.68% |
|  | PZE-109120606 | 9 | N | Other | 13.98 | 13.56 | 16.12 | 15.76 | 18.17 | 18.19 | 37 | 220 | 14.40% | 85.60% |
|  | PZE-110051617 | 10 | G | A | 13.69 | 12.53 | 16.02 | 14.83 | 18.39 | 17.19 | 204 | 24 | 79.38% | 9.34% |
|  | PZE-101172517 | 1 | A | C | 13.43 | 13.76 | 15.81 | 15.79 | 18.32 | 18.1 | 92 | 160 | 35.80% | 62.26% |
|  | PZE-102140170 | 2 | A | G | 13.54 | 13.84 | 15.79 | 15.86 | 18.21 | 18.06 | 201 | 50 | 78.21% | 19.46% |
|  | SYN24938 | 2 | A | G | 13.54 | 13.65 | 15.68 | 15.78 | 18.36 | 18.11 | 36 | 214 | 14.01% | 83.27% |
|  | PZE-102072960 | 2 | A | G | 13.57 | 13.62 | 16.36 | 15.6 | 18.95 | 17.89 | 65 | 185 | 25.29% | 71.98% |
|  | PZE-105090079 | 5 | G | A | 13.47 | 13.62 | 15.55 | 15.87 | 18.35 | 18.11 | 34 | 169 | 13.23% | 65.76% |
|  | PZE-106050102 | 6 | G | A | 13.56 | 13.64 | 15.65 | 16.14 | 18.19 | 18.2 | 167 | 80 | 64.98% | 31.13% |
|  | PZE-102085765 | 2 | C | A | 13.42 | 13.67 | 15.65 | 15.89 | 18.09 | 18.25 | 74 | 178 | 28.79% | 69.26% |
|  | SYN5985 | 1 | G | A | 14.04 | 13.3 | 15.9 | 15.66 | 18.35 | 18.01 | 106 | 144 | 41.25% | 56.03% |
|  | SYN35339 | 1 | A | G | 13.22 | 13.71 | 15.68 | 15.81 | 18 | 18.23 | 49 | 202 | 19.07% | 78.60% |
|  | PZE-101121408 | 1 | G | A | 13.56 | 13.76 | 15.78 | 15.93 | 18.21 | 18.14 | 197 | 56 | 76.65% | 21.79% |
|  | SYN6428 | 1 | A | G | 13.41 | 13.64 | 15.8 | 15.8 | 18.09 | 18.18 | 60 | 190 | 23.35% | 73.93% |
| SBS | SYN11882 | 1 | G | A | 26.32 | 20.52 | 28.47 | 25.05 | 36.78 | 31.57 | 26 | 223 | 10.12% | 86.77% |
|  | PZE-101092091 | 1 | A | C | 25.86 | 19.51 | 26.69 | 24.97 | 35.96 | 31.33 | 55 | 189 | 21.40% | 73.54% |
|  | PZE-101187823 | 1 | A | G | 20.64 | 21.25 | 28.07 | 23.92 | 32.54 | 31.85 | 66 | 179 | 25.68% | 69.65% |
|  | SYN2035 | 1 | A | G | 26 | 19.27 | 27.76 | 24.37 | 32.99 | 31.69 | 65 | 187 | 25.29% | 72.76% |
|  | PZE-101088556 | 1 | A | G | 22.21 | 13.4 | 25.9 | 18.29 | 33 | 17.23 | 201 | 5 | 78.21% | 1.95% |
|  | PZE-102065424 | 2 | G | A | 21.81 | 20.93 | 28.8 | 23.74 | 33.3 | 31.64 | 75 | 174 | 29.18% | 67.70% |
|  | PZE-102087014 | 2 | G | A | 21.77 | 20.84 | 28.28 | 23.68 | 34.99 | 30.53 | 84 | 156 | 32.68% | 60.70% |
|  | PZE-102131796 | 2 | C | A | 22.11 | 17.02 | 26.34 | 21.31 | 32.5 | 29.85 | 109 | 40 | 42.41% | 15.56% |
|  | PZE-102153450 | 2 | G | A | 29.16 | 20.35 | 28.66 | 24.93 | 39.94 | 31.43 | 23 | 223 | 8.95% | 86.77% |
|  | PZE-102181251 | 2 | A | C | 21.87 | 20.85 | 27.9 | 24.02 | 32.7 | 31.93 | 91 | 150 | 35.41% | 58.37% |
|  | PZE-103065478 | 3 | A | G | 23.01 | 19.14 | 26.5 | 23.71 | 34.42 | 29.61 | 119 | 113 | 46.30% | 43.97% |
|  | SYN31353 | 3 | A | G | 22.05 | 18.76 | 26.71 | 21.94 | 33.17 | 30.04 | 168 | 48 | 65.37% | 18.68% |
|  | PZE-103014981 | 3 | G | A | 23.19 | 20.61 | 29.34 | 23.6 | 32.04 | 32.29 | 70 | 177 | 27.24% | 68.87% |
|  | PZE-103049024 | 3 | A | G | 26.78 | 20.7 | 29.1 | 24.91 | 38.56 | 31.51 | 32 | 207 | 12.45% | 80.54% |
|  | SYN12523 | 3 | A | C | 22.99 | 17.81 | 27 | 22.58 | 33.98 | 29.62 | 157 | 88 | 61.09% | 34.24% |
|  | PZE-104024876 | 4 | A | G | 21.84 | 20.68 | 26.97 | 23.87 | 34.63 | 30.28 | 106 | 142 | 41.25% | 55.25% |
|  | PZE-104066962 | 4 | A | C | 22.97 | 19.38 | 27.03 | 23.71 | 33.31 | 30.65 | 139 | 112 | 54.09% | 43.58% |
|  | PZE-104121323 | 4 | G | A | 24.44 | 20.06 | 26.27 | 25.11 | 34.31 | 31.71 | 66 | 183 | 25.68% | 71.21% |
|  | PZE-105036664 | 5 | A | C | 22.74 | 17.27 | 27.11 | 21.32 | 33.5 | 29.69 | 166 | 82 | 64.59% | 31.91% |
|  | PZE-105036968 | 5 | C | A | 22.05 | 18.06 | 26.26 | 21.8 | 33.35 | 27.99 | 198 | 51 | 77.04% | 19.84% |
|  | PZE-105040138 | 5 | A | G | 22.18 | 17.53 | 26.87 | 20.83 | 32.28 | 31.78 | 185 | 64 | 71.98% | 24.90% |
|  | PZE-105182674 | 5 | A | G | 22.62 | 20.48 | 26.27 | 24.98 | 35.57 | 30.12 | 93 | 155 | 36.19% | 60.31% |
|  | PZE-106078845 | 6 | C | A | 21.9 | 19.41 | 26.18 | 22.4 | 33.64 | 27.27 | 191 | 53 | 74.32% | 20.62% |
|  | PUT-163a-149009086-759 | 6 | A | G | 22.39 | 20.31 | 27.93 | 23.39 | 33.79 | 30.62 | 120 | 126 | 46.69% | 49.03% |
|  | PZE-106062991 | 6 | G | A | 24.7 | 18.86 | 27.36 | 23.81 | 34.39 | 30.84 | 88 | 158 | 34.24% | 61.48% |
|  | PZE-107017680 | 7 | A | C | 31.06 | 20.49 | 31.45 | 24.8 | 40.5 | 31.63 | 18 | 238 | 7.00% | 92.61% |
|  | PZE-107072420 | 7 | A | G | 22.31 | 17.55 | 25.85 | 23.89 | 33.36 | 28.73 | 191 | 52 | 74.32% | 20.23% |
|  | PUT-163a-89252658-4661 | 7 | A | C | 25.24 | 21.17 | 34.08 | 25.09 | 49.01 | 31.57 | 8 | 237 | 3.11% | 92.22% |
|  | PZE-107063605 | 7 | G | A | 27.3 | 19.3 | 30.95 | 23.59 | 34.76 | 31.29 | 56 | 191 | 21.79% | 74.32% |
|  | PZE-107038182 | 7 | C | G | 21.89 | 18.98 | 26.13 | 22.7 | 32.9 | 29.75 | 194 | 54 | 75.49% | 21.01% |
|  | SYN36037 | 7 | N | Other | 24.46 | 20.5 | 27.25 | 24.92 | 33.55 | 31.94 | 49 | 208 | 19.07% | 80.93% |
|  | PZE-108089807 | 8 | A | G | 21.14 | 21.26 | 26.08 | 22.63 | 33.61 | 28.46 | 178 | 73 | 69.26% | 28.40% |
|  | PZE-108044852 | 8 | A | G | 23.29 | 19.42 | 27.25 | 23.85 | 33.26 | 31.38 | 99 | 146 | 38.52% | 56.81% |
|  | PZE-109015492 | 9 | A | G | 22.76 | 17.19 | 26.31 | 23.01 | 33.53 | 28.99 | 174 | 70 | 67.70% | 27.24% |
|  | PZE-109085537 | 9 | G | A | 24.11 | 20.13 | 28.9 | 23.81 | 35.03 | 31.26 | 65 | 180 | 25.29% | 70.04% |
| RPR | PZE-101084058 | 1 | A | G | 42.53 | 36.3 | 42.77 | 38.88 | 46.92 | 42.64 | 145 | 104 | 56.42% | 40.47% |
|  | PZE-101124014 | 1 | A | G | 41.95 | 38.79 | 42.89 | 39.95 | 46.41 | 44.44 | 82 | 165 | 31.91% | 64.20% |
|  | PZE-101203731 | 1 | G | A | 40.78 | 36.33 | 42.13 | 36.99 | 45.91 | 41.2 | 195 | 47 | 75.88% | 18.29% |
|  | SYN20044 | 1 | G | A | 40.5 | 36.45 | 41.79 | 37.49 | 45.98 | 40.9 | 207 | 44 | 80.54% | 17.12% |
|  | SYN25989 | 1 | A | C | 40.39 | 38.69 | 41.31 | 40.1 | 46.2 | 43.88 | 118 | 108 | 45.91% | 42.02% |
|  | SYN3142 | 1 | G | A | 40.24 | 39.76 | 43.04 | 40.56 | 47.21 | 44.61 | 43 | 211 | 16.73% | 82.10% |
|  | PZE-101187823 | 1 | A | G | 42.2 | 38.81 | 44.53 | 39.66 | 47.62 | 43.99 | 66 | 179 | 25.68% | 69.65% |
|  | PZE-101000754 | 1 | A | G | 42.89 | 39.36 | 44.5 | 40.47 | 47.42 | 44.63 | 36 | 217 | 14.01% | 84.44% |
|  | PZE-101044692 | 1 | Other | N | 40.36 | 36.26 | 41.41 | 38.75 | 45.44 | 42.08 | 226 | 31 | 87.94% | 12.06% |
|  | PZE-101117214 | 1 | A | G | 42.41 | 39.03 | 44.44 | 40.04 | 48.18 | 44.21 | 52 | 190 | 20.23% | 73.93% |
|  | PZE-101174158 | 1 | G | A | 41.13 | 38.69 | 41.95 | 40.32 | 47.04 | 43.55 | 105 | 143 | 40.86% | 55.64% |
|  | SYN1129 | 2 | A | G | 43.22 | 38.76 | 43.79 | 40.18 | 48.79 | 43.86 | 54 | 194 | 21.01% | 75.49% |
|  | PZE-102054526 | 2 | A | C | 42.87 | 37.91 | 43.27 | 39.58 | 48.85 | 42.87 | 76 | 162 | 29.57% | 63.04% |
|  | PZE-102061736 | 2 | C | A | 41.02 | 39.11 | 43.13 | 39.85 | 45.99 | 44.48 | 91 | 157 | 35.41% | 61.09% |
|  | PZE-102144430 | 2 | G | A | 42.55 | 35.7 | 42.93 | 38.78 | 47.01 | 42.22 | 126 | 101 | 49.03% | 39.30% |
|  | SYN5616 | 2 | G | A | 41.33 | 38.23 | 43.46 | 39.01 | 46.44 | 43.58 | 117 | 130 | 45.53% | 50.58% |
|  | PZE-102123949 | 2 | G | A | 40.61 | 37.15 | 41.47 | 39.75 | 45.34 | 44.02 | 190 | 61 | 73.93% | 23.74% |
|  | PZE-102097169 | 2 | A | G | 44.98 | 39.34 | 45.08 | 40.57 | 50.41 | 44.32 | 25 | 221 | 9.73% | 85.99% |
|  | PZE-102100623 | 2 | G | A | 42.38 | 38.22 | 42.99 | 39.97 | 47.85 | 43.27 | 94 | 151 | 36.58% | 58.75% |
|  | PZE-102101730 | 2 | A | G | 41.86 | 36.89 | 42.62 | 39.03 | 47.34 | 41.67 | 137 | 86 | 53.31% | 33.46% |
|  | PZE-102155296 | 2 | A | G | 40.49 | 39.47 | 42.39 | 40.59 | 46.92 | 44.33 | 57 | 191 | 22.18% | 74.32% |
|  | PZE-103027778 | 3 | A | G | 40.42 | 37.14 | 41.55 | 38.94 | 45.72 | 42.13 | 203 | 49 | 78.99% | 19.07% |
|  | PZE-103031244 | 3 | A | C | 42.6 | 39.11 | 43.74 | 40.5 | 48.49 | 44.23 | 43 | 208 | 16.73% | 80.93% |
|  | SYN23234 | 3 | C | A | 42.19 | 37.52 | 43.08 | 39.27 | 47.47 | 42.77 | 120 | 131 | 46.69% | 50.97% |
|  | PZA03154.4 | 3 | A | G | 40.9 | 37.38 | 42.37 | 37.88 | 45.61 | 43.44 | 185 | 67 | 71.98% | 26.07% |
|  | SYN31353 | 3 | A | G | 40.81 | 37.67 | 42.44 | 37.88 | 46.35 | 41.99 | 168 | 48 | 65.37% | 18.68% |
|  | PZE-103146956 | 3 | A | G | 40.93 | 38.31 | 42.55 | 39.28 | 45.95 | 43.88 | 141 | 105 | 54.86% | 40.86% |
|  | SYN1513 | 4 | C | A | 41 | 37.08 | 42.01 | 38.49 | 46.07 | 42.63 | 172 | 76 | 66.93% | 29.57% |
|  | PZE-104021283 | 4 | A | G | 40.65 | 30.61 | 41.59 | 34.69 | 45.56 | 37.93 | 233 | 17 | 90.66% | 6.61% |
|  | PZE-104047241 | 4 | G | A | 40.53 | 34.35 | 41.31 | 40.03 | 45.53 | 41.48 | 225 | 28 | 87.55% | 10.89% |
|  | SYN18772 | 4 | A | G | 41.37 | 37.61 | 42.28 | 39.52 | 46.82 | 42.56 | 144 | 103 | 56.03% | 40.08% |
|  | SYN37893 | 4 | A | C | 40.9 | 35.18 | 41.87 | 37.89 | 45.71 | 42.33 | 194 | 49 | 75.49% | 19.07% |
|  | PUT-163a-29945048-1805 | 4 | C | G | 42.31 | 37.13 | 43.3 | 38.76 | 46.98 | 43.13 | 137 | 102 | 53.31% | 39.69% |
|  | PZE-104072828 | 4 | A | G | 41.43 | 39.46 | 41.74 | 40.95 | 47.29 | 44.18 | 67 | 181 | 26.07% | 70.43% |
|  | PZE-105036664 | 5 | A | C | 40.87 | 37.28 | 42.09 | 38.74 | 46.4 | 42.33 | 166 | 82 | 64.59% | 31.91% |
|  | SYN2371 | 5 | G | A | 40.43 | 39.41 | 42.19 | 40.69 | 46.93 | 43.94 | 103 | 143 | 40.08% | 55.64% |
|  | PZE-105042796 | 5 | C | A | 41.71 | 38.61 | 43.61 | 39.46 | 46.8 | 43.88 | 79 | 165 | 30.74% | 64.20% |
|  | PZE-105046797 | 5 | G | A | 40.73 | 39.03 | 43.03 | 39.84 | 45.94 | 44.17 | 101 | 110 | 39.30% | 42.80% |
|  | SYN22989 | 6 | G | A | 42.07 | 37.45 | 42.89 | 39.17 | 46.9 | 43.09 | 119 | 129 | 46.30% | 50.19% |
|  | PZE-107061039 | 7 | A | C | 44 | 36.87 | 43 | 39.28 | 48.63 | 42.07 | 82 | 118 | 31.91% | 45.91% |
|  | SYN34663 | 7 | G | A | 42.66 | 37.38 | 43.09 | 39.25 | 47.32 | 43.01 | 119 | 122 | 46.30% | 47.47% |
|  | PZE-107063605 | 7 | G | A | 42.51 | 38.89 | 45 | 40 | 47.26 | 44.38 | 56 | 191 | 21.79% | 74.32% |
|  | SYN18172 | 8 | C | A | 40.57 | 35.5 | 42.02 | 35.9 | 46.02 | 39.5 | 218 | 38 | 84.82% | 14.79% |
|  | PUT-163a-148961306-579 | 8 | C | G | 43.04 | 37.57 | 43.04 | 39.99 | 46.55 | 44.08 | 103 | 148 | 40.08% | 57.59% |
|  | PZE-108067573 | 8 | G | A | 41.08 | 38.82 | 42.05 | 40.55 | 47.39 | 43.35 | 110 | 139 | 42.80% | 54.09% |
|  | PZE-109022267 | 9 | G | A | 41.64 | 38.45 | 42.73 | 39.77 | 46.36 | 44 | 122 | 128 | 47.47% | 49.81% |
|  | PUT-163a-13381789-203 | 9 | A | T | 39.79 | 38.95 | 41.83 | 37.95 | 45.05 | 44.54 | 201 | 47 | 78.21% | 18.29% |
|  | PZE-109105485 | 9 | A | C | 41.54 | 37.05 | 41.64 | 40.44 | 46.11 | 43.31 | 155 | 88 | 60.31% | 34.24% |
